# Delayed Neurological Manifestation in Krait Bites Despite Anti-snake Venom Therapy

**DOI:** 10.7759/cureus.29849

**Published:** 2022-10-02

**Authors:** Shraddha Sawhney, Keta Vagha, Sham Lohiya, Naman Mishra, Jayant D Vagha, Ashish Varma

**Affiliations:** 1 Medicine, Jawaharlal Nehru Medical College, Datta Meghe Institute of Medical Sciences, Wardha, IND; 2 Department of Pediatrics, Jawaharlal Nehru Medical College, Acharya Vinoba Bhave Rural Hospital, Datta Meghe Institute of Medical Sciences, Wardha, IND; 3 Department of Pediatrics, Jawaharlal Nehru Medical College, Datta Meghe Institute of Medical Sciences, Wardha, IND

**Keywords:** case report, anti-snake venom, ptosis, respiratory paralysis, common krait, snake bite

## Abstract

A severe medical emergency that poses a life-threatening risk is envenomation from a snake bite. Among the several snake families, krait bites are known to result in neurological symptoms, including ptosis, headache, and sweating. A 12-year-old adolescent boy who had been bitten by a krait appeared in this instance. The patient showed neurological symptoms after receiving anti-snake venom (ASV). He had three rounds of ASV and made a full recovery. To the best of our knowledge, there have not yet been any reports of this kind of delayed neurological signs after a krait bite, despite getting ASV in the adolescent population.

## Introduction

Cobra and krait species belong to the family Elapidae and are the most prevalent venomous snakes in India [1]. Envenomation of these snakes primarily causes neurotoxicity. All muscles, including the muscles of respiration, can experience sudden neuromuscular paralysis due to neurotoxicity, which can even directly damage the neurons [2]. Changes in the clinical presentations of patients with neurotoxic venomous bites have been related to seasonal, regional, and genetic differences within species in the components of snake venoms. In patients who experience a delayed course of symptoms due to snakebite, several factors, such as the dosage of injected venom, the composition of the poison injected, and delayed hospital admission, might be conceivable [3]. According to WHO, snakebite is a severe tropical illness worldwide [4].

Here, we report a case of a 12-year-old adolescent boy who presented with features of neurotoxic venomous snakebites. He was treated with anti-snake venom within 12 hours of the snakebite. He showed partial recovery but again showed relapse of the neurotoxic features, which required prolonged mechanical ventilation. This kind of relapse of the neurotoxic features is rare; therefore, we wish to report this novel case.

## Case presentation

Patient information

**Figure 1:**
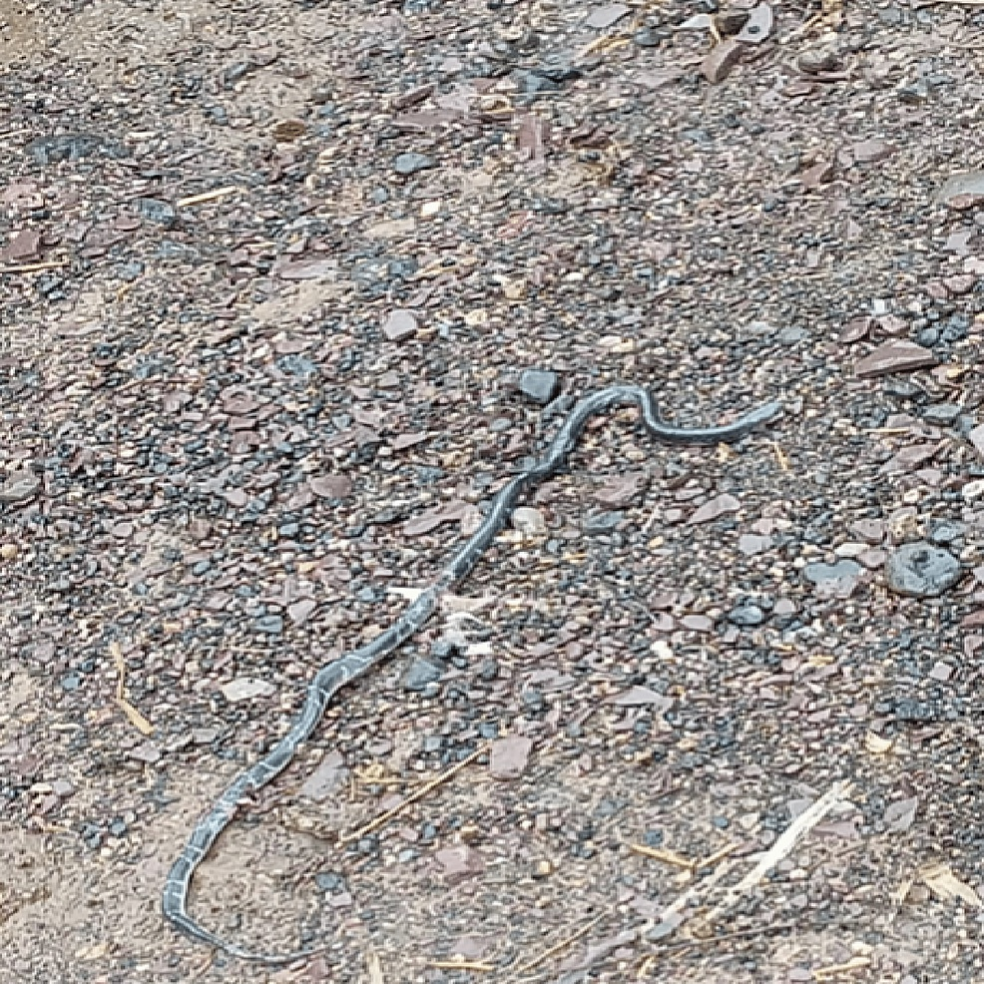
A common Indian krait is shown. Picture captured by S.S.

A 12-year-old adolescent boy was taken to the emergency unit of the primary health care center by his family with a suspected snakebite on his back by a common krait (Figure 1).

**Figure 2:**
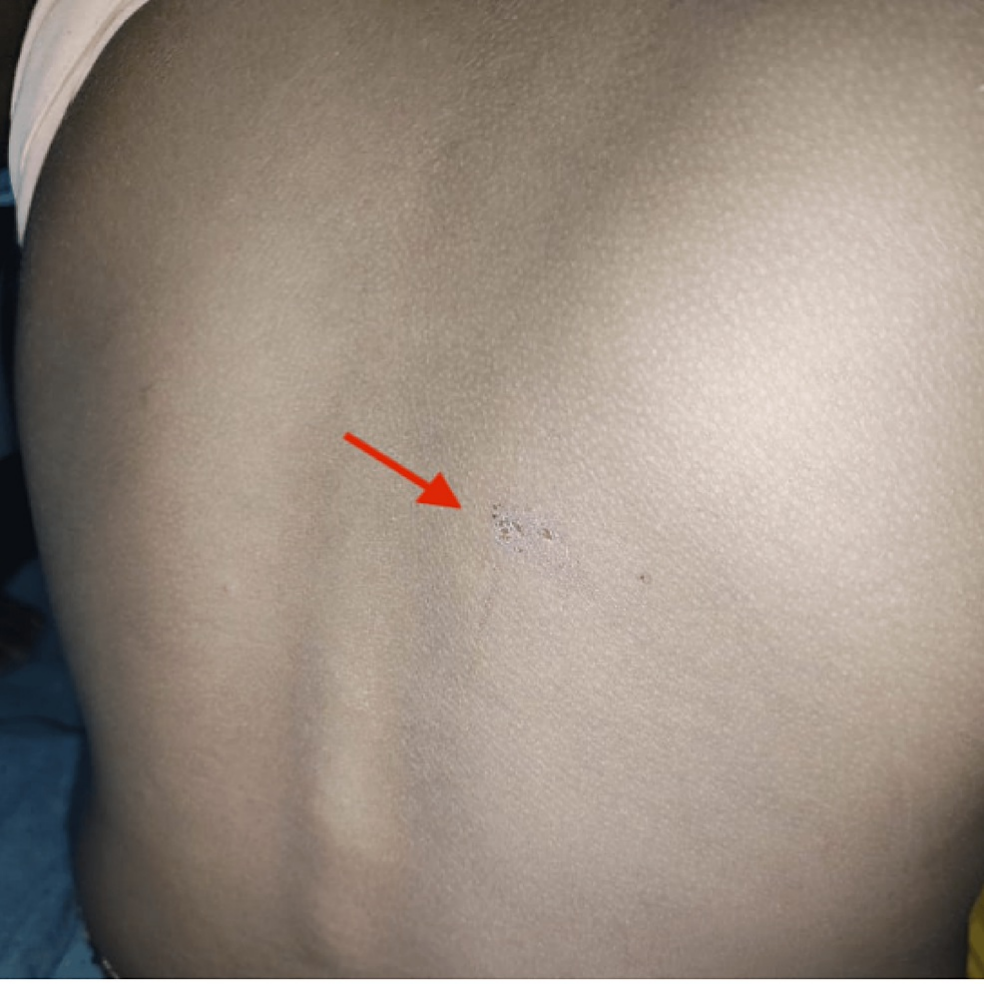
Fang marks in the right infrascapular region are shown. Picture captured by S.S.

He was sleeping when he was bitten by the snake on the back (Figure 2), which he realized due to immense pain at the bite site.

**Figure 3:**
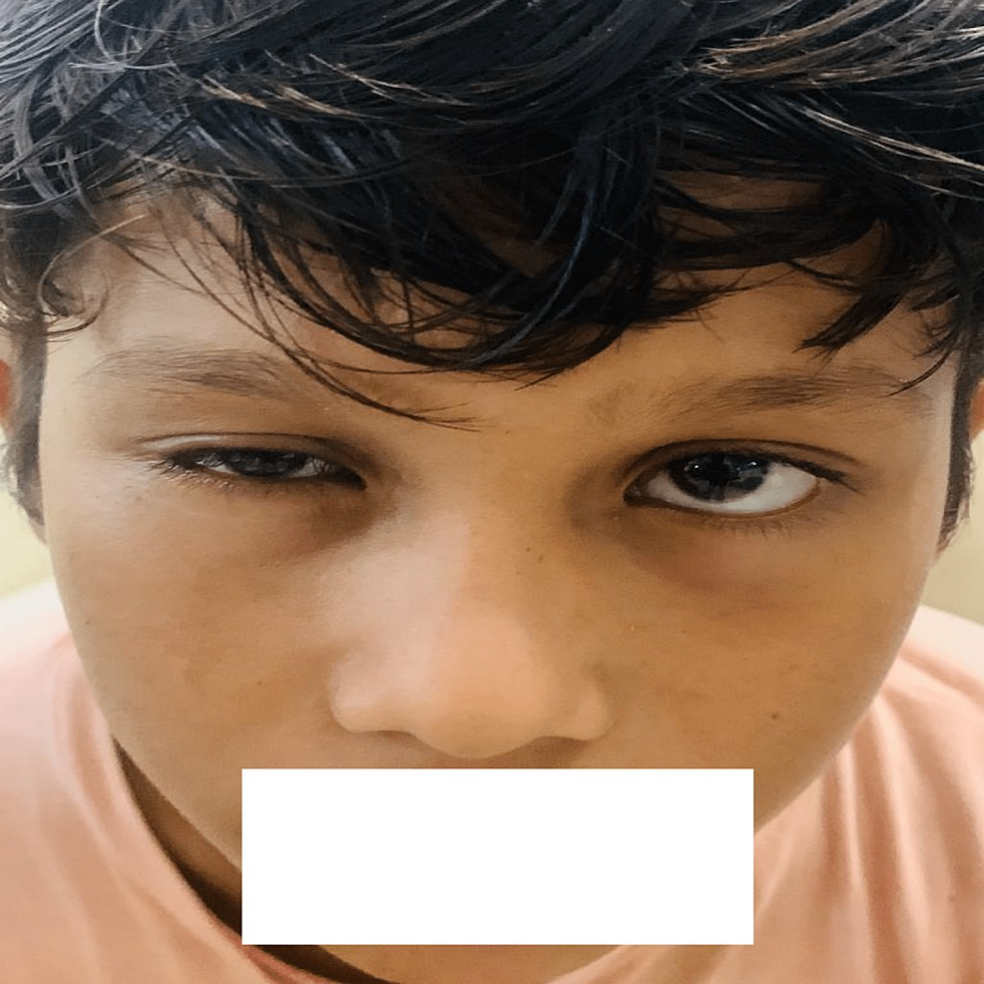
Unilateral ptosis in the left eye is shown. Picture captured by S.S.

He eventually became drowsy when he was being carried to the center. He received 15 vials of anti-snake venom (ASV) within an hour of admission. He gradually developed ptosis after three hours of admission (Figure 3) and then developed respiratory distress; therefore, he was started with supplemental oxygen. However, the respiratory pattern started to deteriorate further; therefore, he was intubated and referred to our tertiary care center in Central India for further treatment. When the child was examined at our emergency unit, he was brought intubated with a bag and tube ventilation.

​​​​​Clinical results

The child was unconscious. His pulse rate was 112 beats per minute, respiratory rate of 36 breaths per minute, a seesaw pattern of breathing, oxygen saturation (SpO2) of 88 on bag and tube ventilation, and a fang mark on the back (Figure 3). The neurological examination showed bilateral ptosis with responsive pupils. Hypotonia and hyporeflexia were noted in all the four limbs, the air entry was equal on both the sides; but he had labored breathing with no other systemic abnormalities. He was shifted to the Pediatric Intensive Care Unit and put on pressure control mode of the mechanical ventilator. He was started with IV fluids and injectable ceftriaxone.

Diagnostic approach

The child's routine blood investigations showed the following readings: hemoglobin, 12 gm/dL; total leucocyte count, 4,600 mm^3^; and platelets, 3.2 lacs/mm^3^. His liver function test, coagulation profile, renal function tests, and 20 minutes whole blood clotting test were normal.

Therapeutic interventions

The child was again administered with six vials of polyvalent ASV (Bharat Serums and Vaccines Limited, Mumbai, India) in normal saline over 30 minutes as there was no improvement in the breathing pattern, ptosis, and the tone of the extremities. He showed slight improvement in form of improved consciousness and diminished ptosis, but the breathing pattern was the same. After 24 hours of the second ASV transfusion, again six more vials of ASV were transfused. After 12 hours of the third transfusion, he started showing improvements in sensorium and the breathing pattern and reduced ptosis and tone of the extremities. As he started improving gradually, there was no further requirement for ASV transfusion; therefore, the mechanical ventilator settings were also eased gradually. On day 7 of his admission, he was weaned off the mechanical ventilator and put on supplemental oxygen; the breathing pattern was still not completely normal, but he was tolerating the change in the treatment well. The bite site did not show necrosis and healed well. 

Outcome of the intervention and follow-up

Gradually, the breathing pattern became normal, and he was weaned off supplemental oxygen. Ptosis was completely improved along with the tone of the limbs, and he could walk with support. For faster recovery, he was advised physiotherapy. On day 13 of his admission, he was discharged, as he showed complete recovery, and was advised for a regular follow-up and continuation of physiotherapy. 

## Discussion

In rural India, snakebites are one of the most common medical emergencies and account for an average of 58,000 fatalities annually [5]. Multiple toxins have been identified in the venom of snakes. The snakes that are most frequently seen in India are the saw-scaled viper (*Daboia russelli*), the common krait (*Bungarus caeruleus*), and the Indian cobra [6]. The krait and king cobra frequently cause neuroparalysis. It is challenging for the patient to get to a health institution in time because most of these snakebites take place in rural locations. As mechanical ventilation is not accessible, the likelihood of high mortality from respiratory arrest is evident.

A common krait can be identified as a long, thin snake. It can measure up to 1.5 m. The upper side of the body is very glossy, with a series of tiny white paired stripes. Krait venom comprises a variety of different neurotoxins of multiple distinct forms. It also has a kappa subunit, which attaches to the neuronal nicotinic acetylcholine receptor at the postsynaptic level in autonomic ganglia cholinergic synapses, in addition to the previously known alpha and beta subunits. Many neurotoxic individuals experience ptosis and extraocular muscle weakness, while a few experience weakness in the respiratory muscle. It is unclear what factors contribute to respiratory muscle weakness in some people. The long-held belief is that it is connected to venom dosage and intensity of envenoming. The effects of neurotoxic substances are reversible, that is, they disappear, either immediately in reaction to antivenom within 30 minutes or after one to seven days.

A 200 mL ASV bolus dose is administered six hours after a 100 mL ASV repeated dose. The same dose is usually administered intravenously to both adults and children, either as a bolus or as a diluted saline solution over 10 to 60 minutes. Neostigmine dosage of 0.04 mg/kg IV and atropine/glycopyrrolate may be continuously infused in situations of neurotoxicity [1]. Phospholipase A2, which is present in the venom of the common krait, degrades the phospholipids present in cell membranes, causing the exhaustion of synaptic vesicles from the motor nerve endings of skeletal muscle, breakdown of the motor nerve endings, and degeneration of the cytoskeleton of the intramuscular axons [7]. This may explain the axonopathy pattern observed in our patient's nerve conduction examination. Furthermore, this destruction is typically permanent, and the patient's recovery depends on the regeneration of new nerve terminals, which may explain why respiratory paralysis can last for varying lengths of time. In another case report from India, a 46-year-old male developed a delayed neurological recovery post administration of ASV [2]. Similarly, another case of an 18-year-old male showing persistent ptosis and respiratory distress, similar to this patient, was reported [8]. The circulating venom is neutralized with polyvalent ASV. The toxin that has already been bonded is not rendered inactive and continues to damage the cells. This explains why patients do not show a good prognosis with a late administration of ASV. In this approach, prompt and early treatment of ASV may improve the prognosis of patients with snakebite envenomation [9]. There is a paucity of literature regarding delayed neurological manifestations; therefore, the exact cause cannot be reported.

## Conclusions

This clinical report highlights the unprecedented and delayed healing of a patient from a common krait bite. A frequent delay in ASV injection might cause a delay in recovery. In addition, the instance may shed some light on the long-term effects of phospholipase A2, a neurotoxin found in snake venom. This enzyme can destroy nerve terminals instead of merely interfering with neuromuscular transmission. However, this might be the first case of an adolescent facing delayed neurological manifestation like ptosis after receiving ASV therapy following a common krait bite. Therefore, it is imperative to monitor the patient for at least a week following ASV administration.

## References

[1] Ghosh S, Mukhopadhyay P, Chatterjee T (2022). Management of snake bite in India. J Assoc Physicians India.

[2] Manappallil RG (2017). Delayed neurological manifestation in viper bite despite anti-snake venom therapy. Int J Adv Med.

[3] Ranawaka UK, Lalloo DG, de Silva HJ (2013). Neurotoxicity in snakebite—the limits of our knowledge. PLoS Negl Trop Dis.

[4] (2019). Snakebite envenoming -- A strategy for prevention and control WHO. https://www.who.int/publications/i/item/9789241515641.

[5] Suraweera W, Warrell D, Whitaker R (2020). Trends in snakebite deaths in India from 2000 to 2019 in a nationally representative mortality study. Elife.

[6] Law AD, Agrawal AK, Bhalla A (2014). Indian common krait envenomation presenting as coma and hypertension: a case report and literature review. J Emerg Trauma Shock.

[7] Dixon RW, Harris JB (1999). Nerve terminal damage by beta-bungarotoxin: its clinical significance. Am J Pathol.

[8] Kadirvelu G, Gnanamoorthy K, Suthakaran P (2022). Fight or flee: an interesting case of snakebite with delayed recovery. Cureus.

[9] Bell DJ, Wijegunasinghe D, Samarakoon S (2010). Neurophysiological findings in patients 1 year after snake bite induced neurotoxicity in Sri Lanka. Trans R Soc Trop Med Hyg.

